# Human Amniotic Membrane Positioning in the Surgical Treatment of Temporomandibular Joint Degenerative Disorder

**DOI:** 10.1155/2019/6037191

**Published:** 2019-03-03

**Authors:** Luca Guarda-Nardini, Diletta Trojan, Giulia Montagner, Elisa Cogliati, Matteo Bendini, Daniele Manfredini

**Affiliations:** ^1^Section of Dentistry and Maxillofacial Surgery, Treviso Hospital, Treviso, Italy; ^2^Treviso Tissue Bank Foundation, Treviso, Italy; ^3^Section of Neuroradiology, Treviso Hospital, Treviso, Italy; ^4^School of Dentistry, Department of Neuroscience, University of Padova, Padova, Italy

## Abstract

**Background:**

Temporomandibular joint (TMJ) arthritis is a degenerative pathology that may cause pain and dysfunction. Nonsurgical therapy is the traditional treatment of TMJ diseases but if ineffective, TMJ surgery can be performed and may include arthroplasty with interposition of autograft. The encouraging results reported with the use of human amniotic membrane (HAM) in different surgical fields have highlighted its potential, but approaches providing the positioning of HAM within the intra-articular space of arthritic TMJs have never been investigated.

**Case Presentation:**

A 48-year-old woman was presented with limited mouth opening and pain with palpation at the left joint. A severe TMJ degeneration was diagnosed, and a surgical treatment was necessary. In the present case report, the authors describe the application of a cryopreserved HAM patch within the joint space as a disc-replacing film during major surgeries for discectomy and arthroplasty. Three months after the intervention, the patient reported an overall improvement in chewing efficiency as well as the absence of pain.

**Conclusions:**

According to the regenerative effects of HAM, the design of trials on the topic should be encouraged for its possible inclusion within the field of TMJ disease practice.

## 1. Introduction

Temporomandibular disorders (TMDs) are the most frequent orofacial pain condition having multifactorial aetiology and affecting the intra-articular structures of the TMJs or the masticatory muscles [1]. They comprised a number of signs and symptoms: masticatory muscle and/or TMJ pain, articular sound during mandibular movement, and abnormalities of the mouth opening path, such as reduction or deviation of the range of joint motion. TMJ degenerative disorders have a complicated aetiology; a decreased adaptive capacity in the articulating structures or sustained physical stress may cause the degenerative remodeling of TMJs [2]. The traditional treatment of TMJ diseases is primarily based on nonsurgical options such as physical therapy, nonsteroidal anti-inflammatory drugs, or arthrocentesis with lubrification [3–5], while surgical treatment of TMJ diseases is only rarely indicated.

The choice of the treatment intervention depends primarily on the diagnosis. Structural disorders (e.g., overt or occult trauma, primary or secondary tumors, degenerative disorders, and infective diseases) [6–11] must be differentiated from nonstructural pain syndromes (e.g., neuropathic pain, myofascial pain, and psychological amplification) [12–14]. Amongst the former, surgery-demanding conditions must be clearly identified.

Within these premises, TMJ arthroplasty with interposition of autograft (i.e., dermis, muscle-temporal fascia, and ear cartilage) is one of the primary surgical treatments [15–17]; however, the use of an autologous tissue might be associated with the morbidity of the second surgical site.

Human amniotic membrane (HAM) is the innermost layer of the fetal membranes with anti-inflammatory, antimicrobial, antifibrotic immunomodulatory properties, epithelialization and cell differentiation-modulating capacities, and low immunogenicity [18–20]. The extensive use and the encouraging results reported with the use of HAM in different surgical fields including maxillofacial surgery have highlighted its potential [21, 22].

The placenta is usually sourced from donors undergoing caesarean sections and processed shortly after retrieval. The HAM is carefully detached from the chorion and rinsed with sterile saline solution to remove residual blood. The membrane is flattened on a nitrocellulose membrane filter (Merck Millipore), with its stromal/mesenchymal side facing down, in contact with the filter. Afterwards, the HAM is immersed in a cocktail of antibiotics including vancomycin 100 *μ*g/ml (Hospira), meropenem 200 *μ*g/ml (Fresenius Kabi Italia), and gentamicin 200 mg/ml (Fisiopharma) at +4°C for 24 h in sterile conditions, validated for human tissues [23]. HAM was cut in 3 × 3 cm^2^ patches and cryopreserved. Microbiological analyses are performed at several stages throughout the process and only HAMs without microbial contamination were considered suitable for implants. The so-obtained tissue is positioned within the joint space.

The positioning of human amniotic membrane (HAM) within the intra-articular space of arthritic TMJs has never been investigated, but increasing amount of evidence highlights the potential positive effects of HAM on a number of surgical conditions, even included the interpositional arthroplasty for TMJ ankylosis [24, 25].

Based on these premises, HAM positioning within the intra-articular space of temporomandibular joints with severe inflammatory-degenerative disorders was suggested [26]. The purpose of this article is to report a case of patient with a diagnosis of TMD who underwent major surgery with the application of a cryopreserved HAM patch.

## 2. Case Presentation

**Figure 1:**
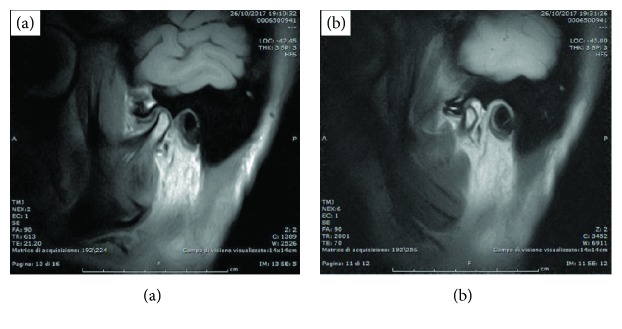
MRI performed in closed (a) and open mouth (b) position during the first visit (T0).

A 48-year-old female came to our observation due to a limitation in mouth opening range. She also reported crepitus sounds at the left TMJ as well as pain, exacerbated by function (e.g., chewing) and increasing in intensity over the past three months. Clinical assessment showed a limited mouth opening (i.e., 22 mm) and pain with palpation at the left joint and all the main masticatory muscles, more severe on the left side. At the first appointment (T0), mandible manipulation was performed to achieve a forced opening of about 40 mm. A magnetic resonance (MRI) was prescribed to assess the disc-condyle relationship as the possible source of limitation in mouth opening and to gather some pictorial evaluation of the presence of joint effusion (Figure 1). Despite the clinical suspicion of TMJ arthritis, computerized tomography was not prescribed at this stage due to the expected low impact on treatment planning decisions.

MRI showed a regularly shaped condyle, with an anteriorized disc at closed mouth. At the maximum mouth opening, the condylar translation is reduced and the disc is not recaptured. Joint effusion of severe entity is also present. A conservative approach to provide pain relief and to manage muscle tension was provided, based on counseling, a home program of self-exercise and a stabilization appliance to wear at night. After three months, symptoms improved only partially, with a reduction of pain with muscle palpation but a steady pain at the left joint.

Based on that, a cycle of five arthrocentesis plus viscosupplementation with hyaluronic acid (Sinovial, IBSA) has been performed weekly. Clinical data has been recorded at each time point before each injection and 15 days after the last one (Table 1).

**Figure 2:**
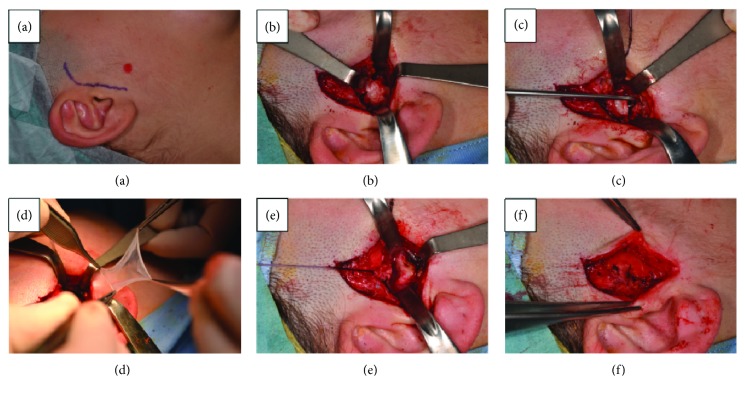
(a–f) condyle remodelling and meniscectomy of TMJ with HAM graft.

After one month from the last arthrocentesis plus viscosupplementation, the patient still showed some pain and, more important, still felt a limitation in the unassisted mouth opening and right laterotrusion. A diagnosis of TMJ intermittent locking on the left side was thus performed, and given the difficulties to stabilize clinical symptoms and mouth opening with the usual conservative approaches, the patient was planned for a surgical removal of the TMJ disc with concurrent HAM positioning (Figure 2).

TMJ surgery provided condyle remodelling and discectomy (Figures 2(a)–2(c)), after which a HAM patch is positioned within the intra-articular space (Figure 2(d)), and stratified stitching is performed to avoid postoperatory scars (Figures 2(e) and 2(f)).

Three months after the intervention, the patient showed no negative exitus or postsurgical side effects. Jaw range of motion was increasing, both as for unassisted mouth opening (38 mm) and right laterotrusion (8 mm). The patient reported an overall improvement in chewing efficiency as well as the absence of pain (Table 2).

**Figure 3:**
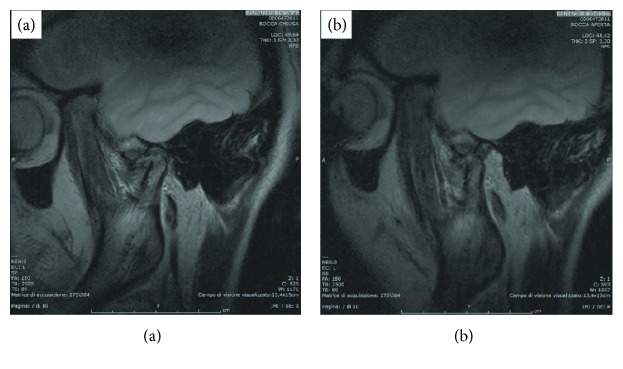
MRI at the five-month follow-up. Closed (a) and open mouth position (b) showing the HAM patch. The HAM patch covers the condyle head; the translation is improved.

Five months after surgery, a new MRI was performed (Figure 3). The images showed the physiological excursion of the TMJ condyle during mouth opening (Figure 3(b)).

## 3. Discussion

This case report demonstrates that in the surgical treatment of TMJ arthrosis, the interposition of HAM in the intra-articular space resulting from an arthroplasty intervention represents a promising solution. In fact, three months after the intervention, the patient reported the absence of pain, an increased masticatory capacity, and an improvement of the range of mouth opening.

Since the etiopathogenesis and symptoms of TMJ diseases are variable, several different treatments are adopted. In rare selected cases, surgical treatments are necessary, including meniscectomy, condylectomy, and arthroplasty with interposition of autologous tissues [15–17].

The benefits of HAM positioning in TMJs with severe inflammatory-degenerative disorders could be related with its anti-inflammatory and antimicrobial properties and low immunogenicity [18–20]. In addition, thanks to its properties, it has been demonstrated that the application of HAM to wounds markedly reduces patients' experienced pain intensity [27]. Moreover, amniotic membrane is able to reduce postoperative adhesion [28].

Within the limits of the present case report, a promising suggestion concerning the use of HAM for TMJ surgery has been provided. Case series providing a longer follow-up should be encouraged, as well as the assessment of less invasive strategies for HAM positioning. In particular, future clinical trials might aim at comparing the effectiveness of HAM with respect to standard interpositional surgical intervention. Imaging techniques trying to assess the intra-articular changes associated with the use of human membrane are also recommended for a better comprehension of the course of degenerative disorders in patients undergoing this treatment.

## Figures and Tables

**Table 1:** Clinical data collected before each injection (T0-T4) and 15 days after the last injection.

Parameters	T0	T1	T2	T3	T4	15 days after the last injection
Masticatory capacity (scores from 0 to 10)^∗^	3	3	5	7	7	7
Chewing-related pain (scores from 0 to 10)^∗∗^						
min	0	0	0	0	0	0
max	6	9	6	6	6	6
Phonation-related pain (scores from 0 to 10)^∗∗^						
min	0	0	0	0	0	0
max	8	8	8	1	5	5
Rest-related pain (scores from 0 a 10)^∗∗^						
min	0	0	0	0	0	0
max	0	7	0	0	3	3
Level of functional limitation^§^	4	4	1	1	3	3
Efficacy evaluation^∗∗∗^	-	1	2	3	1	1
Tolerability evaluation^∗∗∗^	-	2	3	3	3	3
Mouth-opening ability						
Spontaneous	23 mm	23 mm	30 mm	33 mm	25 mm	25 mm
Forced	23 mm	42 mm	35 mm	33 mm	25 mm	27 mm
Right laterality	0	0	0	10 mm	10 mm	10 mm
Protusion	5 mm	5 mm	5 mm	5 mm	3 mm	3 mm
Left laterality	10 mm	10 mm	10 mm	10 mm	10 mm	10 mm

^∗^Scores from 0 = inability to chew and only the consumption of semiliquid foods is possible to 10 = optimal chewing capacity of any type of food. ^∗∗^NRS scores from 0 = no pain to 10 = intolerable pain. ^§^0 = none; 1 = tolerable; 2 = moderate; 3 = intense; 4 = serious. ^∗∗∗^0 = light; 1 = tolerable; 2 = moderate; 3 = good; 4 = excellent.

**Table 2:** Three-month follow-up assessment. Clinical data.

Parameters	3 months after surgery
Masticatory capacity (scores from 0 to 10)^∗^	8
Chewing-related pain (scores from 0 to 10)^∗∗^	
min	0
max	0
Phonation-related pain (scores from 0 to 10)^∗∗^	
min	0
max	0
Rest-related pain (scores from 0 to 10)^∗∗^	
min	0
max	2
Level of functional limitation^§^	2
Efficacy evaluation^∗∗∗^	3
Tolerability evaluation^∗∗∗^	3
Mouth-opening ability	
Spontaneous	38 mm
Forced	38 mm

^∗^Scores from 0 = inability to chew and only the consumption of semiliquid foods is possible to 10 = optimal chewing capacity of any type of food. ^∗∗^NRS scores from 0 = no pain to 10 = intolerable pain. ^§^0 = none; 1 = tolerable; 2 = moderate; 3 = intense; 4 = serious. ^∗∗∗^0 = light; 1 = tolerable; 2 = moderate; 3 = good; 4 = excellent.

## References

[1] Romero-Reyes M., Uyanik J. M. (2014). Orofacial pain management: current perspectives. *Journal of Pain Research*.

[2] Tanaka E., Detamore M. S., Mercuri L. G. (2008). Degenerative disorders of the temporomandibular joint: etiology, diagnosis, and treatment. *Journal of Dental Research*.

[3] McNeely M. L., Armijo Olivo S., Magee D. J. (2006). A systematic review of the effectiveness of physical therapy interventions for temporomandibular disorders. *Physical Therapy*.

[4] Manfredini D., Piccotti F., Guarda-Nardini L. (2010). Hyaluronic acid in the treatment of TMJ disorders. A systematic review of the literature. *CRANIO®*.

[5] Moldez M. A., Camones V. R., Ramos G. E., Padilla M., Enciso R. (2018). Effectiveness of intra-articular injections of sodium hyaluronate or corticosteroids for intracapsular temporomandibular disorders: a systematic review and meta-analysis. *Journal of Oral & Facial Pain and Headache*.

[6] Miyauchi K., Sano K., Nagai M. (2006). Occult fractures of articular eminence and glenoid fossa presenting as temporomandibular disorder: a case report. *Oral Surgery, Oral Medicine, Oral Pathology, Oral Radiology, and Endodontology*.

[7] Paparo F., Massarelli M., Cordeschi R., Sciannameo V., Spallaccia F. (2016). Chondromatosis of the temporomandibular joint as a consequence of persistent long-lasting joint dysfunction: late diagnosis of a rare occurrence. *Journal of Craniofacial Surgery*.

[8] Lohiya S., Dillon J. (2016). Septic arthritis of the temporomandibular joint—unusual presentations. *Journal of Oral and Maxillofacial Surgery*.

[9] Park H. J., Kim B. C., Choi E. J., Samayoa S. R., Kim H. J. (2014). Tuberculosis of the temporomandibular joint: a case of misdiagnosis. *Journal of Oral & Facial Pain and Headache*.

[10] Guarda-Nardini L., Stellini E., Di Fiore A., Manfredini D. (2017). A rare case of misdiagnosed silent lung cancer with solitary metastasis to the temporomandibular joint condyle. *Journal of Oral & Facial Pain and Headache*.

[11] Emanuelsson J., Allen C. M., Rydin K., Sjöström M. (2017). Osteoblastoma of the temporal articular tubercle misdiagnosed as a temporomandibular joint disorder. *International Journal of Oral and Maxillofacial Surgery*.

[12] Prisco L., Ganau M., Bigotto F., Zornada F. (2011). Trigeminal neuralgia: successful antiepileptic drug combination therapy in three refractory cases. *Drug, Healthcare and Patient Safety*.

[13] Guarda-Nardini L., Piccotti F., Ferronato G., Manfredini D. (2012). Myositis ossificans traumatica of the temporalis muscle: a case report and diagnostic considerations. *Oral and Maxillofacial Surgery*.

[14] Horswell B. B., Sheikh J. (2018). Evaluation of pain syndromes, headache, and temporomandibular joint disorders in children. *Oral and Maxillofacial Surgery Clinics of North America*.

[15] Bayat M., Badri A., Moharamnejad N. (2009). Treatment of temporomandibular joint ankylosis: gap and interpositional arthroplasty with temporalis muscle flap. *Oral and Maxillofacial Surgery*.

[16] Holmlund A., Lund B., Weiner C. K. (2013). Mandibular condylectomy with osteoarthrectomy with and without transfer of the temporalis muscle. *British Journal of Oral and Maxillofacial Surgery*.

[17] Yazdani J., Ali Ghavimi M., Pourshahidi S., Ebrahimi H. (2010). Comparison of clinical efficacy of temporalis myofascial flap and dermal graft as interpositional material in treatment of temporomandibular joint ankylosis. *Journal of Craniofacial Surgery*.

[18] Inge E., Talmi Y. P., Sigler L., Finkelstein Y., Zohar Y. (1991). Antibacterial properties of human amniotic membranes. *Placenta*.

[19] Hao Y., Ma D., Hwang D., Kim W., Zhang F. (2000). Identification of antiangiogenic and antiinflammatory proteins in human amniotic membrane. *Cornea*.

[20] Koizumi N. J., Inatomi T. J., Sotozono C. J., Fullwood N. J., Quantock A. J., Kinoshita S. (2000). Growth factor mRNA and protein in preserved human amniotic membrane. *Current Eye Research*.

[21] Kothari C. R., Goudar G., Hallur N., Sikkerimath B., Gudi S., Kothari M. C. (2012). Use of amnion as a graft material in vestibuloplasty: a clinical study. *British Journal of Oral and Maxillofacial Surgery*.

[22] Ragazzo M., Trojan D., Spagnol L., Paolin A., Guarda Nardini L. (2018). Use of amniotic membrane in the treatment of patients with BRONJ: two case reports. *Journal of Surgical Case Reports*.

[23] Serafini A., Riello E., Trojan D. (2016). Evaluation of new antibiotic cocktails against contaminating bacteria found in allograft tissues. *Cell and Tissue Banking*.

[24] Akhter M., Ahmed N., Arefin M. R., Sobhan M.-U., Molla M. R., Kamal M. (2016). Outcome of amniotic membrane as an interpositional arthroplasty of TMJ ankylosis. *Oral and Maxillofacial Surgery*.

[25] Bauer F., Hingsammer L. M., Wolff K. D., Kesting M. R. (2013). Temporomandibular joint arthroplasty with human amniotic membrane: a case report. *Eplasty*.

[26] Guarda-Nardini L., Trojan D., Paolin A., Manfredini D. (2017). Management of temporomandibular joint degenerative disorders with human amniotic membrane: hypothesis of action. *Medical Hypotheses*.

[27] Mermet I., Pottier N., Sainthillier J. M. (2007). Use of amniotic membrane transplantation in the treatment of venous leg ulcers. *Wound Repair and Regeneration*.

[28] Walker C. T., Godzik J., Kakarla U. K., Turner J. D., Whiting A. C., Nakaji P. (2018). Human amniotic membrane for the prevention of intradural spinal cord adhesions: retrospective review of its novel use in a case series of 14 patients. *Neurosurgery*.

